# Effect of AMH on primordial follicle populations in mouse ovaries and human pre-pubertal ovarian xenografts during doxorubicin treatment

**DOI:** 10.3389/fcell.2024.1449156

**Published:** 2024-08-27

**Authors:** Xi Wei, Briet D. Bjarkadottir, Devi Nadjaja, Sairah Sheikh, Muhammad Fatum, Sheila Lane, Suzannah A. Williams

**Affiliations:** ^1^ Nuffield Department of Women’s and Reproductive Health, Women’s Centre, John Radcliffe Hospital, University of Oxford, Oxford, United Kingdom; ^2^ Oxford Fertility, Institute of Reproductive Sciences, Oxford, United Kingdom; ^3^ Department of Paediatric Oncology and Haematology, Children’s Hospital Oxford, Oxford University Hospitals NHS Foundation Trust, Oxford, United Kingdom

**Keywords:** anti-Müllerian hormone, doxorubicin, fertility preservation, primordial follicles, ovarian tissue, human ovarian xenograft, ovary, follicle

## Abstract

**Introduction:**

Survival rates of the childhood cancer patients are improving, however cancer treatments such as chemotherapy may lead to infertility due to loss of the primordial follicle (PMF) reserve. Doxorubicin (DXR) is a gonadotoxic chemotherapy agent commonly used in childhood cancers. Anti-Müllerian Hormone (AMH) has been reported to have a protective effect on the mouse ovarian reserve against DXR *in vivo*. However, whether AMH can prevent PMF loss in conjunction with DXR in human ovarian tissue *in vivo* has not been determined.

**Methods:**

In order to investigate this, we first established an optimum dose of DXR that induced PMF loss in cultured mouse ovaries and investigated the efficacy of AMH on reducing DXR-induced PMF loss in mice *in vitro*. Second, we investigated the effects of DXR on pre-pubertal human ovarian tissue and the ability of AMH to prevent DXR-induced damage comparing using a mouse xenograft model with different transplantation sites.

**Results:**

Mouse ovaries treated with DXR *in vitro* and *in vivo* had reduced PMF populations and damaged follicle health. We did not observe effect of DXR-induced PMF loss or damage to follicle/stromal health in human ovarian cortex, this might have been due to an insufficient dose or duration of DXR. Although AMH does not prevent DXR-induced PMF loss in pre-pubertal and adult mouse ovaries, in mouse ovaries treated with higher concentration of AMH *in vitro*, DXR did not cause a significant loss in PMFs. This is the first study to illustrate an effect of AMH on DXR-induced PMF loss on pre-pubertal mouse ovaries. However, more experiments with higher doses of AMH and larger sample size are needed to confirm this finding.

**Discussion:**

We did not observe that AMH could prevent DXR-induced PMF loss in mouse ovaries *in vivo*. Further studies are warranted to investigate whether AMH has a protective effect against DXR in xenotransplanted human ovarian tissue. Thus, to obtain robust evidence about the potential of AMH in fertility preservation during chemotherapy treatment, alternative AMH administration strategies need to be explored alongside DXR administration to fully interrogate the effect of DXR and AMH on human xenografted tissues.

## Introduction

Despite an increased long-term survival rate for children and young patients diagnosed with cancer, chemotherapy treatment can result in a number of side effects of which premature ovarian insufficiency (POI) due to loss of the primordial follicle reserve is one of the most distressing (Sklar et al., 2006; Hudson, 2010; Smith et al., 2010; Jayasinghe et al., 2018). For pre-pubertal girls, ovarian tissue cryopreservation is the only fertility preservation option, however it is invasive and currently considered experimental with just two live births from reimplanted tissue that was cryopreserved prior to puberty (Demeestere et al., 2015; Matthews et al., 2018). In recent years there has been a growing interest in developing novel non-invasive methods of fertility preservation (Spears et al., 2019), particularly for young patients or patients with haematological malignancies for whom current fertility preservation methods are either unavailable or risky (Yasmin et al., 2018).

Doxorubicin (DXR) is a commonly used chemotherapeutic agent used for up to 50% of all cancers either alone or in combination with other chemotherapeutic agents (Roti Roti et al., 2014). Clinical studies have shown that the risk of women developing permanent amenorrhea treated with a regimen including DXR widely ranges from less than 20% to over 80% depending on age, pre-chemotherapy ovarian reserve and specific treatment regimen (Lee et al., 2006). The exact mechanism behind DXR-induced POI remains unclear. Animal studies have demonstrated a significant reduction in the population of ovarian follicles, including primordial, following DXR treatment *in vitro* and *in vivo* (Morgan et al., 2013; Roti Roti et al., 2012; Lopes et al., 2020). Recently it has been suggested that the gonadotoxic effect of DXR in mice may occur through both primordial follicle atresia and accelerated activation (Wang et al., 2019). However, evidence of the effect of DXR on human follicles remains limited.

Several agents are currently being investigated to attenuate DXR-induced primordial follicle loss such as Dexrazoxane, Sphingosine-1-phosphate, Bortezomib, Resveratrol and Anti-Müllerian Hormone (AMH; Roti Roti and Salih, 2012; Kropp et al., 2015; Roti Roti et al., 2014; Li et al., 2014; Kano et al., 2017; Herrero et al., 2023). Of these candidate protective molecules, AMH is the only natural hormone generated within the human body. AMH has been suggested for use as a therapeutic agent for Müllerian duct origin cancers (La Marca and Volpe, 2007). AMH acts via pathways unrelated to most chemotherapy agents (Pieretti-Vanmarcke et al., 2006; Rodgers et al., 2021) and does not appear to interfere with the cytotoxic actions of the chemotherapy drug as demonstrated in studies using cyclophosphamide (Roness et al., 2019; Kano et al., 2017), suggesting it could be administered in conjunction with chemotherapy agents without affecting the functional pathway of chemotherapy agents to kill cancer cells.

In the ovary, AMH is generated by the granulosa cells of growing follicles (Blanchard and Josso, 1974; Weenen et al., 2004). Currently there is no robust evidence for AMH localisation to primordial follicles (Pankhurst, 2017), however, AMH receptor 2 has been localised in the pre-granulosa cells of primordial follicles in mouse and human ovaries (Kano et al., 2017; Meinsohn et al., 2021), indicating AMH might suppress follicle activation by preventing pre granulosa cell differentiating. Alternatively, AMH may regulate primordial follicle activation through the phosphatidylinositol 3-kinase (PI3K) pathway (Sonigo et al., 2019; Rosario et al., 2024). Studies have demonstrated that AMH attenuates primordial follicle activation or recruitment in mice *in vitro* and *in vivo* (Durlinger et al., 1999; Durlinger et al., 2002; Gigli et al., 2005; Visser et al., 2007; Park et al., 2014; Hayes et al., 2016) and in cultured and grafted bovine tissue (Gigli et al., 2005; Yang et al., 2017). However, studies in other species including ovine and human have been conflicting with regards to whether AMH causes or suppresses follicle activation (Schmidt et al., 2005; Carlsson et al., 2006; Campbell et al., 2012; Bertoldo et al., 2016; Rocha et al., 2016; Man et al., 2017). Studies have suggested that AMH is able to attenuate Cyclophosphamide-induced primordial follicle loss in mice *in vitro* and *in vivo* (Roness et al., 2015; Roness et al., 2019; Sonigo et al., 2019; Kashi et al., 2023) and more recently, AMH reduced Cyclophosphamide-induced PI3K activation in cultured human ovarian cortex (Rosario et al., 2024). PI3K signalling pathway acts on the primordial follicle activation via the forkhead transcription factor Foxo3 (FOXO3A) (John et al., 2008). However, whether AMH can prevent primordial follicle loss in conjunction with DXR in human ovarian tissue has not been determined.

Here we aimed to study the effects of AMH on DXR-induced primordial follicle loss and damage on follicle health in pre-pubertal human ovarian tissue. We first established an optimum dose of DXR that can induce primordial follicle loss in mouse ovaries *in vitro* and investigated the efficacy of AMH on reducing DXR-induced primordial follicle loss in mice *in vitro* (Part 1). In Part 2, we investigated the effects of DXR on pre-pubertal human ovarian tissue and the ability of AMH to prevent DXR-induced damage comparing using a mouse xenograft model with different transplantation sites. We hypothesize that AMH ameliorates DXR-induced primordial follicle loss in mouse ovaries and human ovarian tissues.

## Materials and methods

### Mice

Mice (breeding trios comprised of CD1 females (6 weeks) and C57BL6 males (8 weeks), and SCID females (9–10 weeks) were housed in ventilated cages with a 12-h light/12-h dark cycle and fed with food and clean water, *ad libitum*. Mice were acclimatised for a minimum of 1 week before mating or xenotransplantation.

### Mouse ovarian culture

Mouse ovaries from postnatal day 6 female pups (generated from crossing CD1 females with C57BL6 males) were dissected into pre-warmed Dulbecco’s phosphate buffered saline (PBS; D8537, Sigma, United Kingdom) supplemented with 1 mg/mL bovine serum albumin (BSA; BPE1600-100, Fisher Scientific, United Kingdom). After isolation, the ovaries were cultured for 6 days at 37°C and 5% CO_2_ on Whatman Nucleopore polycarbonate membranes (WHA110414, Sigma) floating on 1 mL pre-equilibrated culture medium in 24-well culture plates (Corning Costar, United Kingdom). Culture medium was comprised of alpha Minimum Essential Medium (α-MEM; 22571, Sigma) supplemented with 3 mg/mL BSA, 100 IU/mL penicillin and 0.1 mg/mL streptomycin (P0781, Sigma) and 50% was changed every 2 days during the culture period.

To determine the appropriate dose of DXR (Sigma) to be used since different preparations can have different toxicity, DXR was added on the second day of culture for 24 h at concentrations of 50, 100 and 200 ng/mL. On day 3, all ovaries were transferred to a new well containing fresh culture medium and membrane for the remaining 4 days of culture.

After assessing the effect of DXR at different concentrations, DXR at 50 ng/mL was selected to investigate the effects of AMH on DXR-induced primordial follicle loss. Recombinant human AMH (rhAMH) was purchased from R&D Systems (1737-MS-010/CF, United Kingdom). On day 2 of culture, 50 ng/mL DXR was added to the DXR and DXR + AMH groups; this medium was replaced entirely with fresh culture medium after 24 h as described previously. AMH was added to the relevant groups at 50, 100 or 200 ng/mL on days 0, 2 and 4 of culture. The concentrations of AMH were determined based on previous studies (Nilsson et al., 2007; Bertoldo et al., 2016; Rocha et al., 2016; Yang et al., 2017). After 6 days of culture, all mouse ovaries were collected.

### Human tissue samples

Ovarian cortical tissue was obtained from pre-pubertal patients who were having a planned unilateral oophorectomy for fertility preservation and had consented to tissue being used for research. Exclusion criteria included previous chemotherapy treatment and disease diagnosis involving ovarian tissues. Ovarian cortical tissue was cut into strips (1 × 1 × 5 mm pieces), cryopreserved by the Oxford Cell and Tissue Biobank (OCTB) technicians as described (Walker et al., 2021) and stored in vapour phase liquid nitrogen.

### Mice used for xenotransplantation

Female mice with severe combined immune deficiency (SCID; CB17/Icr-PrkdcSCID/IcrIcoCrl; Charles River, United Kingdom) at 9–10 weeks of age were used as hosts for human ovarian cortical tissue grafts.

SCID mice were weighed, ear-clipped and divided into four groups by weight to ensure the groups of mice were comparable: Control (PBS-treated; n = 6), AMH treated group (n = 6), DXR-treated group (n = 6) and DXR + AMH co-treatment group (n = 6).

### Human ovarian cortical strip thawing

Cryovials containing ovarian cortical strips were thawed in a water bath at 30°C for 3 min and then washed to remove the cryoprotectant agents in three solutions containing a decreasing gradient of ethylene glycol (1, 0.5 and 0 M; 324558, Sigma), 0.1 M sucrose (S7903, Sigma) and 10% serum substitute supplement (99193, Irvine Scientific, United Kingdom) in Leibovitz L-15 medium (L5520, Sigma). After thawing, cortical strips were put into fresh L-15 medium at 4°C until transplantation.

**FIGURE 1:**
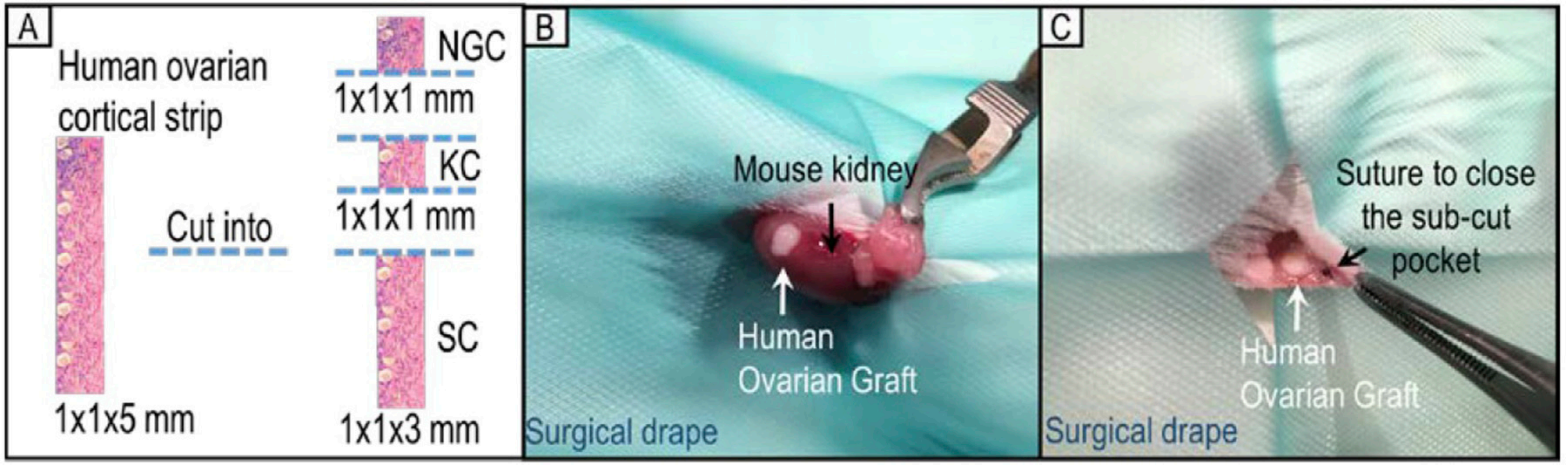
Human ovarian cortical strip division and allocation for xenotransplantation. **(A)** Each strip of ovarian cortical tissue was divided into three pieces for use (NGC: non-grafted control group; KC: kidney capsule; SC: subcutaneous pocket). **(B)** Representative image of xenotransplantation surgery placing one piece of tissue under the kidney capsule of a SCID mouse. **(C)** Representative image of xenotransplantation surgery grafting one piece of human ovarian tissue in a subcutaneous pocket of the same SCID mouse.

It has been observed that follicle development of transplanted ovarian tissue can be influenced by different grafting sites (Dath et al., 2010; Youm et al., 2015). Therefore, two different xenotransplantation sites were investigated and compared in this study. Four strips of ovarian cortical tissue were used from each patient; one for each treatment. Each strip was cut into two 1 × 1 × 1 mm pieces (one for post-thaw non-grafting histological analysis, and the other one for kidney capsule transplantation) and one large (1 × 1 × 3 mm) piece for subcutaneous transplantation (Figure 1A).

### Xenotransplantation surgery

Anaesthesia was induced with 4% isoflurane and 2.5 L/min of oxygen and then maintained at 2.5% isoflurane and 1.5 L/min of oxygen. Analgesic agents, Vetergesic (10%, Ceva Animal Health, United Kingdom) and Matacam (10%, Boehringer Ingelheim, Germany), were administered subcutaneously. Left dorsal and lateral areas of mice were shaved and the exposed skin antiseptically cleaned.

For xenotransplantation under the kidney capsule, a small flank incision of the skin and a further small incision of the body wall were made to access the left kidney. The left kidney was exteriorised and a small pocket was made under the kidney capsule using fine forceps into which a piece of human ovarian tissue was inserted (Figure 1B). The kidney was returned to its normal position and the body wall and skin closed with interrupted sutures using 4-0 Vicryl sutures (W9575, Ethicon, United States).

For subcutaneous xenotransplantation, a small skin incision was made in the left lateral flank of the mouse. A small pocket under the skin was made using fine forceps from the inside, at least 0.5 cm away from the edge of the incision. A graft was inserted into the sub-cutaneous pocket which was closed using a Vicryl 0.7 (6/0) suture (Figure 1C). Skin incisions were closed by interrupted sutures as above.

After surgery, mice were placed in pre-warmed cages with bedding. Soft food (normal food soaked in water) in a petri dish as well as normal food pellets and water in a petri dish were provided. Mice were weighed daily during the first 2 weeks post-surgery and twice a week thereafter.

### Experiment design and drug administration

**FIGURE 2:**
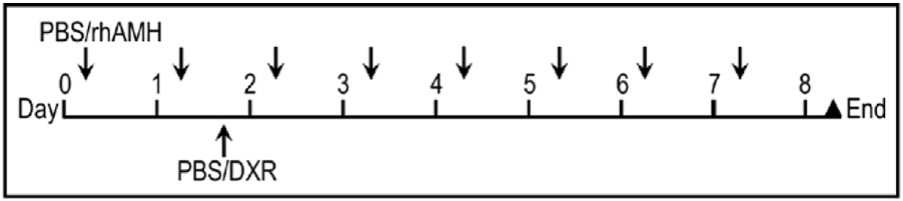
Treatment regimen for administration of anti-Müllerian hormone (AMH) and/or doxorubicin (DXR) to SCID mice (severe combined immunodeficient) containing human ovarian transplants. Ten days after human ovarian tissue was xenotransplanted into SCID mice, animals were intraperitoneally (IP) injected daily with PBS or rhAMH for 8 days with an additional single IP injection of PBS or DXR on the second day.

Ten days after the surgery (Day 0), mice were given a single daily IP injections of 300 ng rhAMH (AMH and DXR + AMH groups) or the same volume of vehicle control (PBS; control and DXR groups) for 8 days (days 0-8; Figure 2). The *in vivo* dose of AMH was determined according to a previous study in mice (Hayes et al., 2016). On the afternoon of day 1, DXR was IP injected into the mice at a dose of 4 mg/kg in the DXR and co-treatment groups and PBS was injected into mice in the Control and AMH groups. This dose was selected as we wanted all hosts to survive and 4 mg/kg was the highest non-lethal dose (Sonowal et al., 2017): a dose of 5 mg/kg in mice results in death within 1 week (Denard et al., 2015). After 8 days of treatment, all mice were sacrificed by cervical dislocation, the human ovarian xenografts were recovered and the mouse ovaries collected.

### Tissue fixation, embedding, sectioning and staining

Cultured and non-cultured mouse ovarian tissue and human ovarian grafts were collected and fixed in 10% neutral buffered formalin (Sigma) for 6 hours (mouse ovarian tissue) or 8 hours (human ovarian tissue), paraffin embedded and serially sectioned at 5 μm. Every 10th section was stained with Haematoxylin (6765008, Fisher Scientific) and Eosin (H&E; HT110316, Sigma), mounted and imaged using a MicroPublisher 5.0 Real-Time Viewing camera (Qimaging, Microscope services Ltd., Canada) attached to a DM2500 Leica microscope (Microscope services Ltd., Woodstock, United Kingdom).

### Follicle morphological assessment, follicle counting and mouse ovarian volume

Only follicles with a visible nucleus were staged as previously described (Lo et al., 2019; Walker et al., 2021). Mouse and human follicles containing an oocyte and surrounded only by flattened pre-granulosa cells were classified as primordial follicles. If the follicle was composed of an oocyte with a layer of mixed flattened pre-granulosa cells and cuboidal granulosa cells, it was classified as transitional. Follicles were defined as primary follicles if they contained one complete layer of cuboidal granulosa cells, and secondary follicles if they contained more than one layer of granulosa cells (Van den Hurk et al., 1997). Follicles with an antrum present were defined as antral follicles. In this study, we specifically focused on primordial follicles, and therefore all follicles beyond the primordial stage were presented as combined data named “growing follicles”.

Follicles stained with H&E were classified as healthy or atretic according to their morphology. A healthy follicle was defined as containing an intact oocyte with a morphologically normal nucleus and cytoplasm, and less than 10% of pyknotic granulosa cells (Tomic et al., 2006). Follicles were considered atretic if they had an oocyte with a pyknotic nucleus and condensed cytoplasm or more than 10% of the granulosa cells contained pyknotic bodies (Tomic et al., 2006). If the total number of granulosa cells was less than 10, e.g., in transitional and primordial follicles, follicles were considered atretic if one pyknotic granulosa cell was present.

Follicles with a visible nucleus were counted after follicle stage classification. Follicles were counted in every 10th section and the Abercrombie correction was applied in order to estimate the total number of follicles (Abercrombie, 1946). The Abercrombie correction corrects for oocyte nucleus area and the thickness of sections. All mouse ovaries and human ovarian grafts were analysed blind. The mouse ovarian area of every 10th section was measured using ImageJ. Ovarian volume was calculated using total ovarian area multiplied by the thickness of section and the interval number. All mouse ovaries were analysed blind.

### TUNEL assay

Four groups of human ovarian xenografts retrieved from the mouse subcutaneous pocket (and their corresponding non-grafted tissue) were used for TUNEL labelling. In addition, TUNEL labelling was performed on the corresponding mouse host ovaries. Different TUNEL assay kits were used for the two species based on prior in-house optimisation experiments. For mouse ovaries, DNA fragmentation was detected using the ApopTag^®^ Peroxidase *in Situ* Apoptosis Detection Kit (S7100, Merck, Germany) according to the manufacturer’s protocol. Labelling was visualised using 3,3′-diaminobenzidine (DAB) (SK-4100, VectorLabs, United Kingdom). For human ovarian xenografts, DNA fragmentation was detected using the FragEL™ DNA Fragmentation Detection Kit (QIA33-1EA, Merck, Germany) according to the manufacturer’s protocol. Labelling was visualised using DAB (supplied in kit). For both mouse and human samples, nuclear counterstaining was performed using Haematoxylin. Cell nuclei that were stained brown were considered TUNEL positive.

For human samples, the percentage of follicles with a TUNEL-positive oocyte and/or more than 10% TUNEL-positive granulosa cells were analysed in sections (n = 3) selected from the top, middle and bottom of a block (Tomic et al., 2006). If the total number of granulosa cells was less than 10, e.g., in transitional and primordial follicles, follicles were recorded if one pyknotic granulosa cell was present. For mouse ovaries, all follicles in a single section were analysed using the same criteria as above. The proportion of TUNEL-positive stroma cells was further assessed in human samples under ×40 magnification in random selected regions of sections (n = 3) selected from the top, middle and bottom of a block of tissue.

### Statistical analyses

All data was analysed using GraphPad Prism version 7.00 (GraphPad Software, La Jolla, CA, United States). Data was tested for normality using the Shapiro-Wilk normality test. One-way ANOVA was applied to determine if different treatments groups generated normally distributed data. If the data of groups were not normally distributed, the Kruskal Wallis test and the Dunn’s multiple comparisons test were used. When comparing two groups, the Mann-Whitney U test was used if the data was not normally distributed. These results are presented as mean ± S.E.M. for multiple comparisons and as mean ± S.D. for a comparison between two independent groups. Significance was considered when *P* < 0.05.

## Results

### Part 1: studies investigating AMH using mouse ovary culture

#### DXR causes primordial follicle loss in cultured mouse ovaries

**FIGURE 3:**
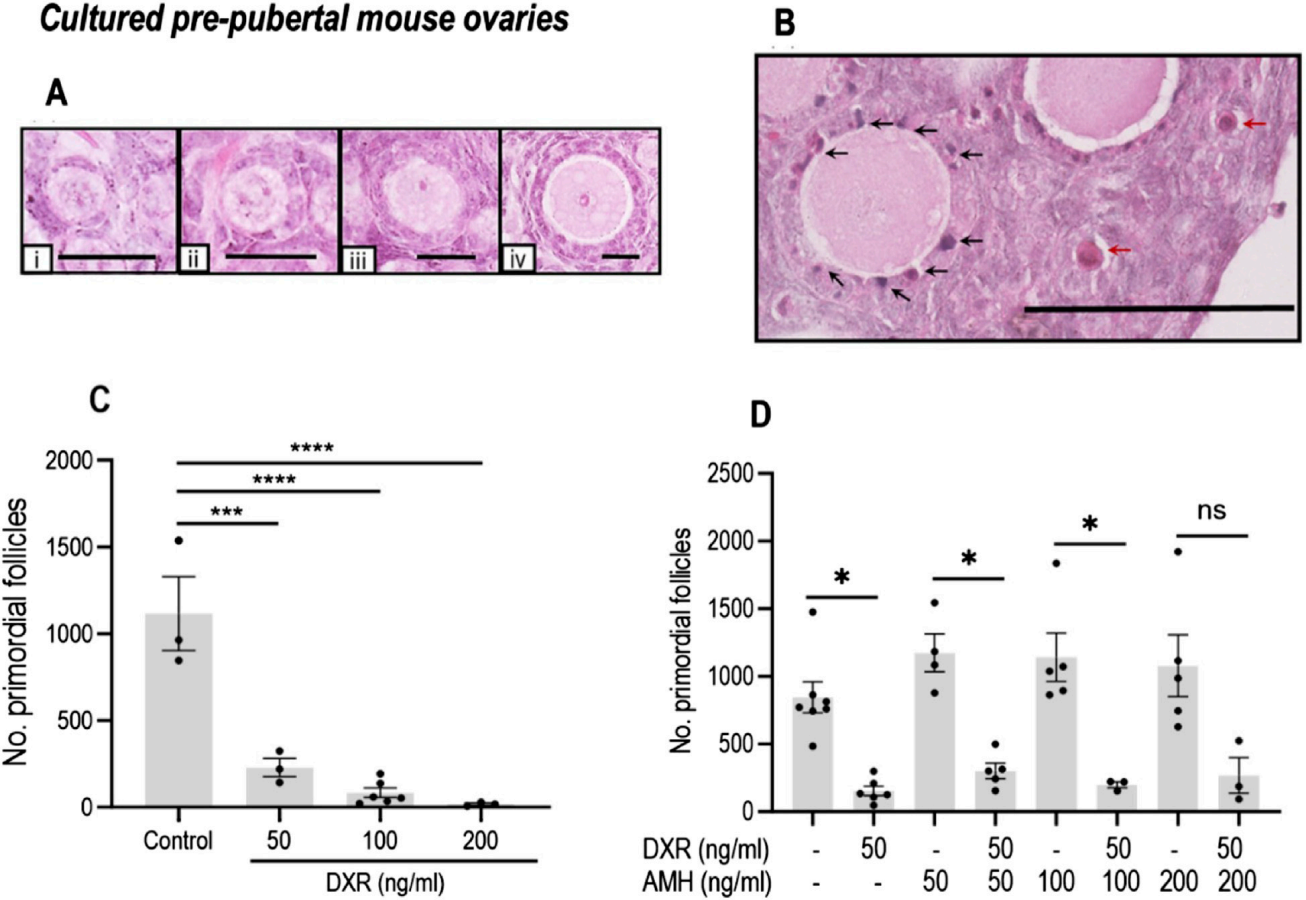
Analysis of mouse ovarian follicles in cultured pre-pubertal ovaries treated with DXR and AMH stained with H and E. **(A)** Representative images of morphologically healthy follicles (scale bar = 25 μm): i: Primordial follicles. ii-iv: Growing follicles (ii: Transitional; iii: Primary; iv: Secondary). **(B)** Unhealthy follicles (scale bar = 100 μm): black arrows denote pyknotic granulosa cells, red arrows denote atretic oocytes. **(C)** Total number of morphologically healthy primordial follicles in mouse ovaries treated with different concentrations of DXR. Values are presented as Mean ± S.E.M. Control n = 3; 50 ng/mL DXR n = 3; 100 ng/mL DXR n = 5; 200 ng/mL DXR n = 3. **(D)** Number of morphologically healthy primordial follicles in mouse ovaries cultured with different concentrations of rhAMH and with or without exposure to DXR. Values represent Mean ± S.E.M ns = not significant, *P < 0.05, ***P < 0.001, ****P < 0.0001.

Based on H&E staining, healthy and unhealthy mouse ovarian follicles showed significant different morphology as illustrated in Figures 3A, B. Total healthy primordial follicle numbers were compared between the control group and DXR-treated. Treatment with 50 ng/mL DXR was sufficient to induce primordial follicle loss (1116.06 ± 213.17 versus 228.31 ± 52.61; *p* < 0.001, Figure 3C). Based on these results, 50 ng/mL DXR was selected for the AMH co-culture experiments.

#### Effect of AMH on primordial follicle numbers in cultured mouse ovaries with or without DXR

As in the previous culture experiment, DXR-treated ovaries had a reduced number of primordial follicles compared to control (153.5 ± 36.07 versus 844.4 ± 114.7; *p* < 0.05, Figure 3D). There was no significant difference in the total number of primordial follicles in AMH-treated ovaries in comparison with the ovaries in the control group (Figure 3D); although all AMH treated groups combined contained 30%–50% more healthy primordial follicles (844.4 ± 114.7 versus 1128 ± 102.9, *p* < 0.05). When comparing the ovaries treated with 50 ng/mL AMH-treated only, co-treatment with DXR induced significant primordial follicle loss (*p* < 0.05, Figure 3D). When ovaries cultured with AMH at 100 ng/mL, co-treatment with DXR still let to a decrease of primordial follicle numbers significantly (*p* < 0.05, Figure 3D). Interestingly, in ovaries treated with DXR and 200 ng/mL AMH, co-treatment with DXR did not lead to a significant decrease of primordial follicle numbers (Figure 3D).

### Part 2: studies investigating AMH using mice xenotransplanted with human ovarian tissue

#### Patient characteristics and human ovary graft recovery

Ovarian cortical strips from six pre-pubertal patients aged 4–12 (mean 8.2 ± 1.4 years) were used for xenotransplantation (Table 1). Cryopreserved-thawed human ovarian cortical strips were xenotransplanted into SCID mice; one piece from each patient was split and transplanted into two sites in the same mouse. Ten days after surgery (day 0), mice were given rhAMH or vehicle control for 8 days (days 0–8; Figure 2) and a single dose of DXR (4 mg/mL) or vehicle control was administered on day 1. After 8 days of treatment, the ovarian xenografts were recovered and the mouse ovaries collected. Graft recovery for Control, AMH, DXR and co-treatment groups is displayed in Table 2 and Supplementary Table S1.

**TABLE 1:** Age and diagnoses of pre-pubertal patient’s tissue (n = 6) used in this study.

Patient no.	Age	Diagnoses
A	4	Beta Thalassemia
B	5	Aplastic anaemia
C	6	Sickle cell disease; progressive cerebrovascular disease
D	10	Severe congenital neutropenia
E	12	Severe aplastic anaemia
F	12	Sickle Cell Disease

**TABLE 2:** Proportion of pre-pubertal human ovarian xenografts retrieved from different transplantation sites in SCID mice.

Sites	Control	AMH	DXR	DXR + AMH
Kidney capsule	6/6	6/6	6/6	4/5
Subcutaneous	6/6	6/6	6/6	5/5

One mouse in the DXR + AMH, group died and was excluded from the study. One kidney capsule graft was not present at recovery in the DXR + AMH, group.

#### AMH treatment does not prevent DXR-induced primordial follicle loss in mice *in vivo*

DXR treatment had a physiological effect on the mice, with DXR-treated mice losing weight after administration of DXR (Supplementary Figure S1A). Mice in the DXR and co-treatment groups also had reduced spleen weight and ovarian volume at the end of the experiment compared to mice in the Control and AMH groups (Supplementary Figure S1B, C).

**FIGURE 4:**
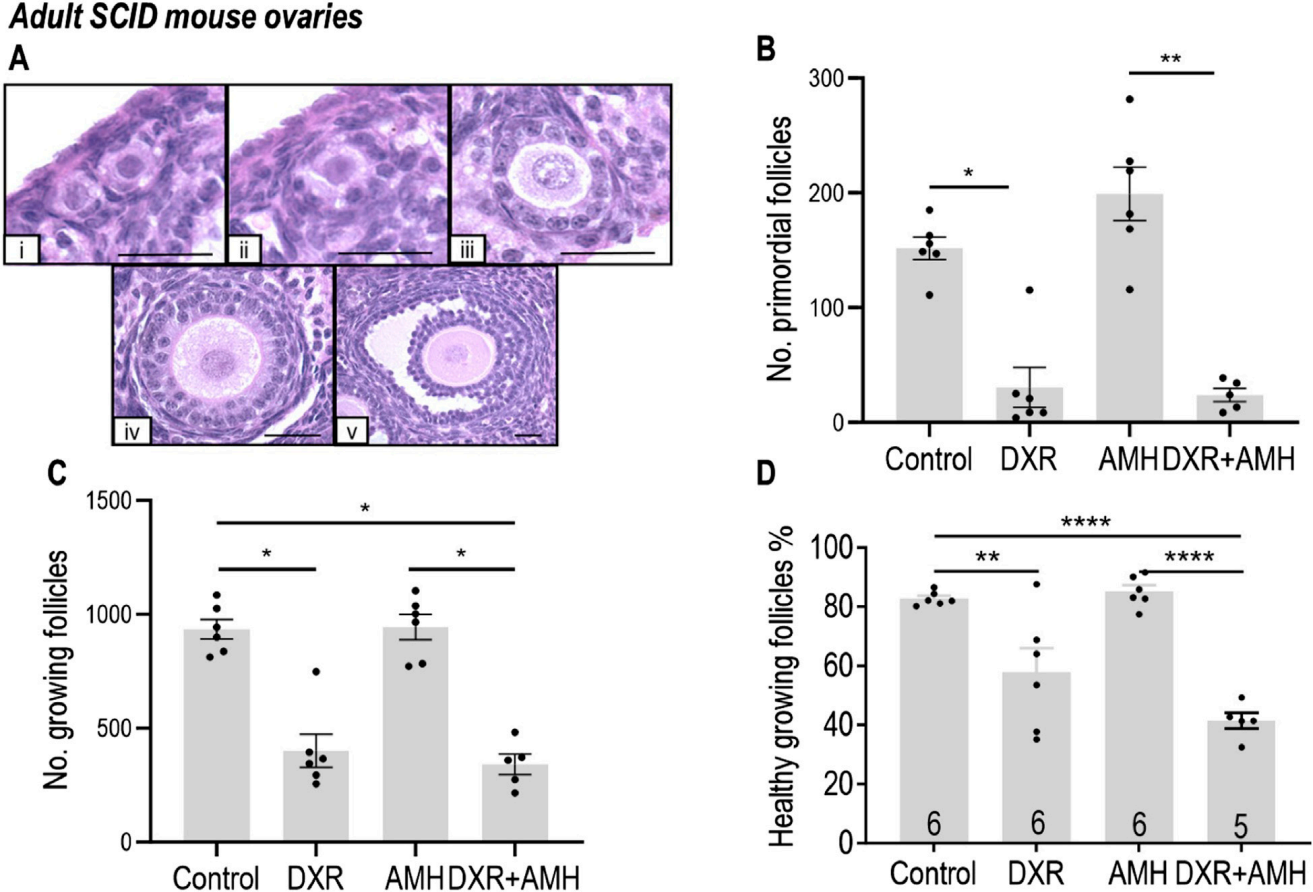
Analysis of mouse ovarian follicles in SCID mice carrying human ovary xenografts treated with AMH and/or DXR stained with H and E. **(A)** Representative images of mouse ovarian follicles (scale bar = 25 μm). **(I)** Primordial follicles. ii-v: Growing follicles (ii: Transitional; iii: Primary; iv: Secondary; v: Antral). **(B)** Number of morphologically healthy primordial follicles in mouse ovaries after 8 days of treatment with PBS, DXR, AMH or co-treatment (DXR + AMH). **(C)** Number of growing follicle numbers in mouse ovaries from the Control, DXR, AMH, and DXR + AMH groups at the end of the experiment. **(D)** Proportion of morphologically healthy growing follicles in mouse ovaries from different groups. The data are presented as Mean ± S.E.M. Number in the base of the column is the sample number. *P < 0.05, **P < 0.01, ****P < 0.0001.

Follicles in the adult mouse ovaries were assessed and defined as primordial or growing (Figure 4A). The total number of primordial follicles was significantly lower in mice in the DXR group compared to the Control group (30.47 ± 17.28 versus 151.8 ± 9.911; *p* < 0.05, Figure 4B). Co-treatment with AMH did not prevent the DXR induced decrease in primordial follicle numbers, with fewer primordial follicles in the mice co-treated with DXR + AMH compared to the mice treated with AMH alone (23.88 ± 5.81 versus 199.0 ± 56.92; *p* < 0.01, Figure 4B). Mice treated with DXR also had fewer growing follicles compared to mice without any DXR treatment (Control or AMH group). Similarly, AMH co-treatment did not prevent the decline in growing follicle numbers induced by DXR (Figure 4C).

The morphological health of growing follicles was assessed. DXR treatment resulted in a significant decrease in growing follicle health, compared to mice without DXR treatment (Control or AMH; Figure 4D). Additional treatment with AMH in the AMH + DXR group did not increase the proportion of healthy follicles compared to DXR alone.

**FIGURE 5:**
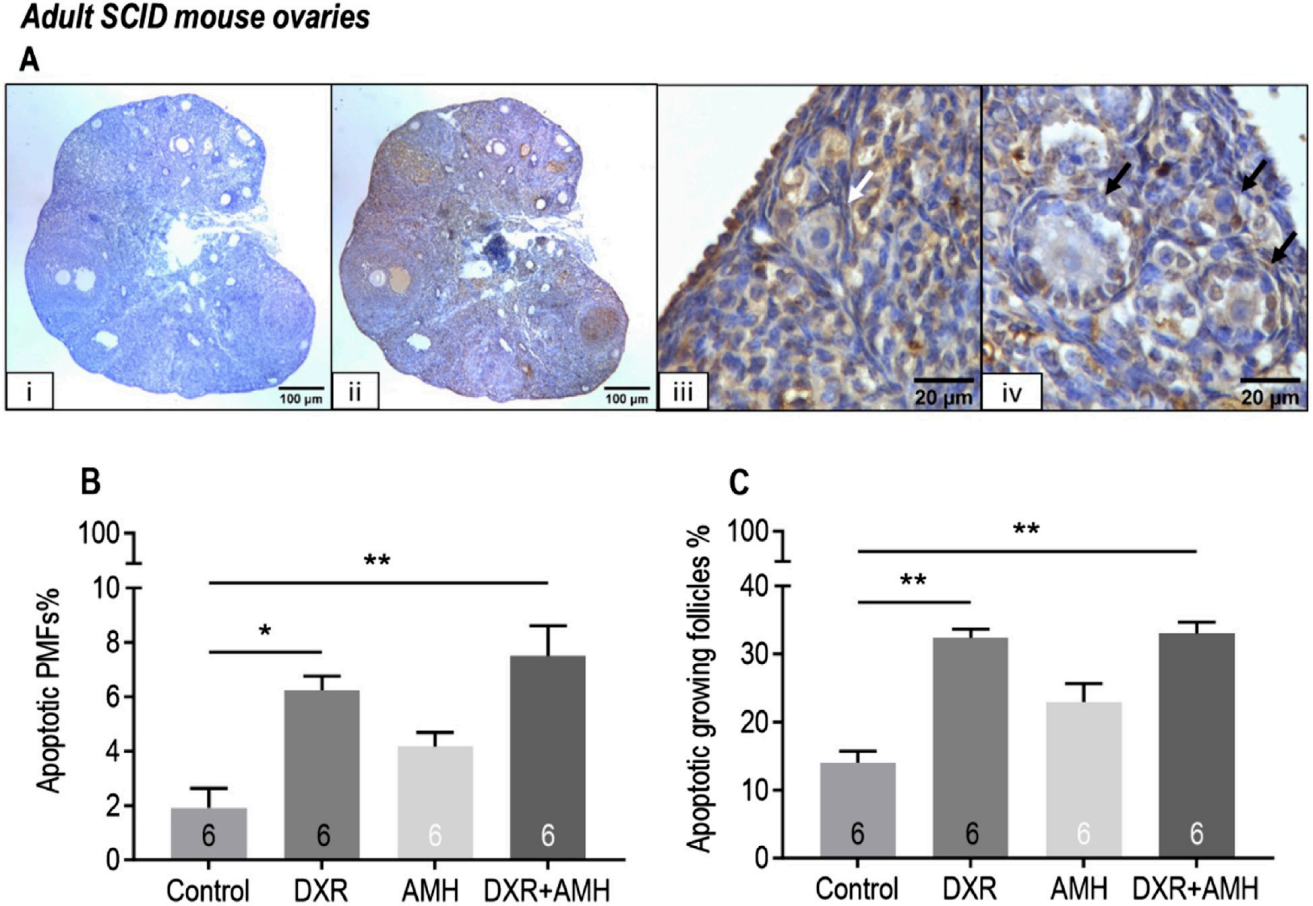
Analysis of mouse apoptotic follicles in SCID mice carrying human ovarian xenografts treated with AMH and/or DXR stained with TUNEL. **(A)** Representative images of TUNEL assay. **(I)** Negative control ovarian section (scale bar = 100 µm), ii: TUNEL-labelled ovarian section (scale bar = 100 µm), iii: TUNEL-negative primordial follicle (white arrow, scale bar = 20 µm), iv: TUNEL-positive growing follicles (black arrows, scale bar = 20 µm). **(B)** Proportion of apoptotic (TUNEL-positive) primordial follicles (PMFs) in mouse ovaries after 8 days of treatment with PBS (Control), DXR, AMH or co-treatment (DXR + AMH). **(C)** Proportion of apoptotic growing follicles in mouse ovaries after 8 days of treatment with PBS (Control), DXR, AMH or co-treatment (DXR + AMH). *P < 0.05, **P < 0.01.

DXR treatment triggered apoptosis in mouse ovarian follicles (Figure 5). There was a significant increase in the proportion of apoptotic primordial follicles for mice treated with DXR (Control: 1.9% ± 0.7%, DXR: 6.2% ± 0.5%, *P* < 0.05) or DXR + AMH (7.5% ± 1.1%, *P* < 0.01; Figure 5B). There was also a significant increase in the proportion of apoptotic growing follicles for mice treated with DXR (Control: 14.0% ± 1.7%, DXR: 32.4% ± 1.3%, *P* < 0.01) or DXR + AMH (33.0% ± 1.7%, *P* < 0.01; Figure 5C). There was no significant difference in TUNEL between the control and AMH only group (Figure 5). AMH co-treatment did not reduce the proportion of apoptotic follicles.

#### Xenotransplanted human ovarian tissue contained healthy growing follicles

**FIGURE 6:**
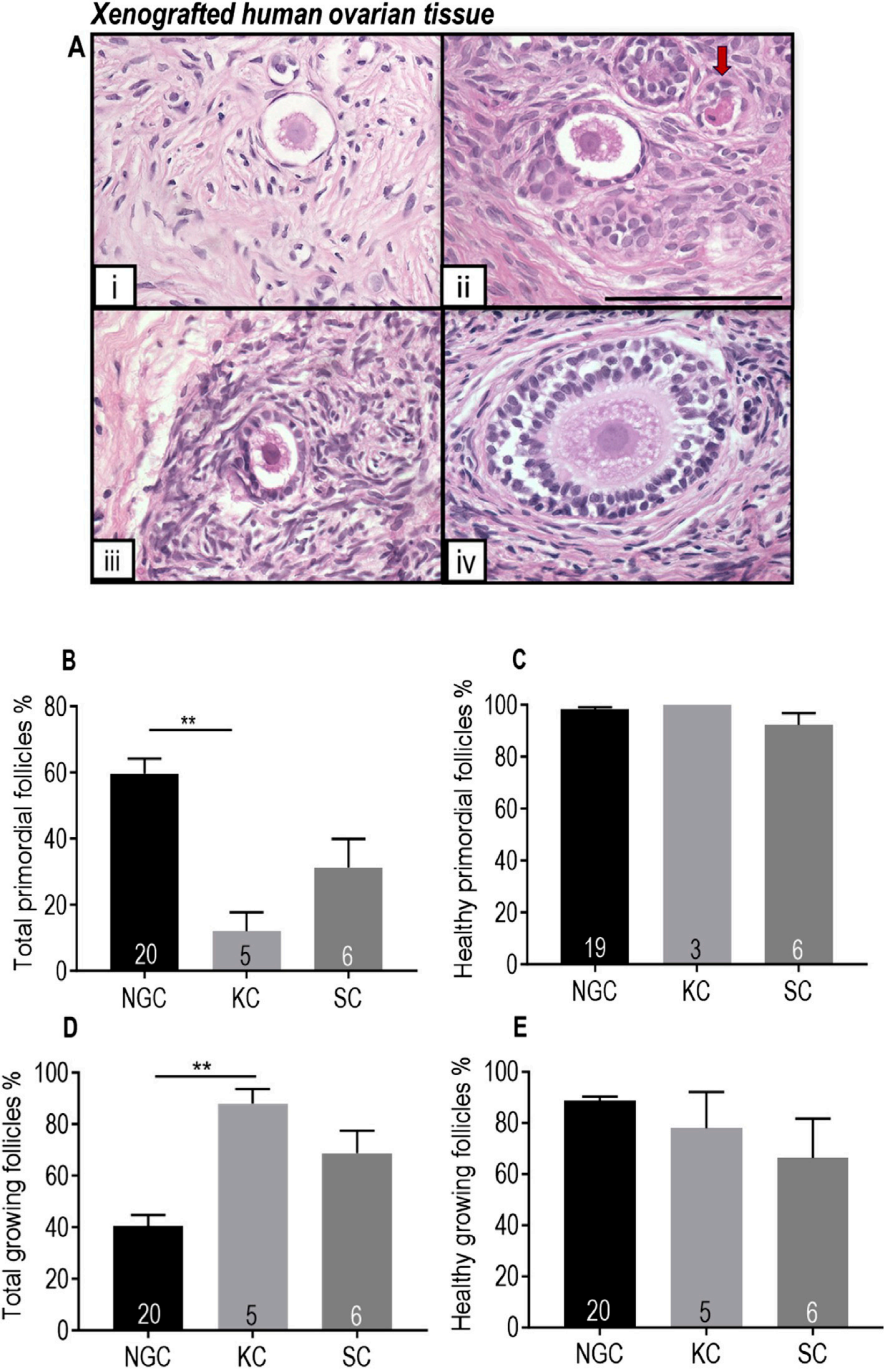
Comparison of human ovarian follicles in xenografted tissue retrieved from the mouse kidney capsule and a subcutaneous pocket in the control group with non-grafted tissue stained with H and E. **(A)** Representative images of follicles at different stages in pre-pubertal human ovarian tissue. i Primordial follicle. ii-iv: Growing follicles (ii: Transitional follicle. iii: Primary follicle. iv: Secondary follicle). Red arrow denotes an unhealthy follicle. Scale bar represents 100 μm. **(B)** Proportion of follicles that were primordial. **(C)** Proportion of primordial follicles that were morphologically healthy. **(D)** Proportion of growing follicles. **(E)** Proportion of morphologically healthy growing follicles. NGC: Non-grafted group. KC: Kidney capsule. SC: Subcutaneous pocket. The data are presented as Mean ± S.E.M. Number in the base of the column is the sample number. **P < 0.01. Total follicle numbers (Mean ± S.E.M.): NGC 335.1 ± 55.3; KC control 76.7 ± 24.62; SC control 278.6 ± 137.6.

Follicles ranging from primordial stage to secondary stage were observed in the human ovarian tissue after xenotransplantation for 18 days (Figure 6A). After xenotransplantation, there was a decrease in the proportion of primordial follicles and an increase in the proportion of growing follicles, indicating development of transplanted human ovarian tissue (Figures 6B, D). There was no significant difference in the morphological health of primordial or growing follicles between grafted tissue and non-grafted controls, indicating good follicle survival (Figures 6C, E).

#### Effect of DXR and AMH on xenotransplanted human ovarian cortical strips

**FIGURE 7:**
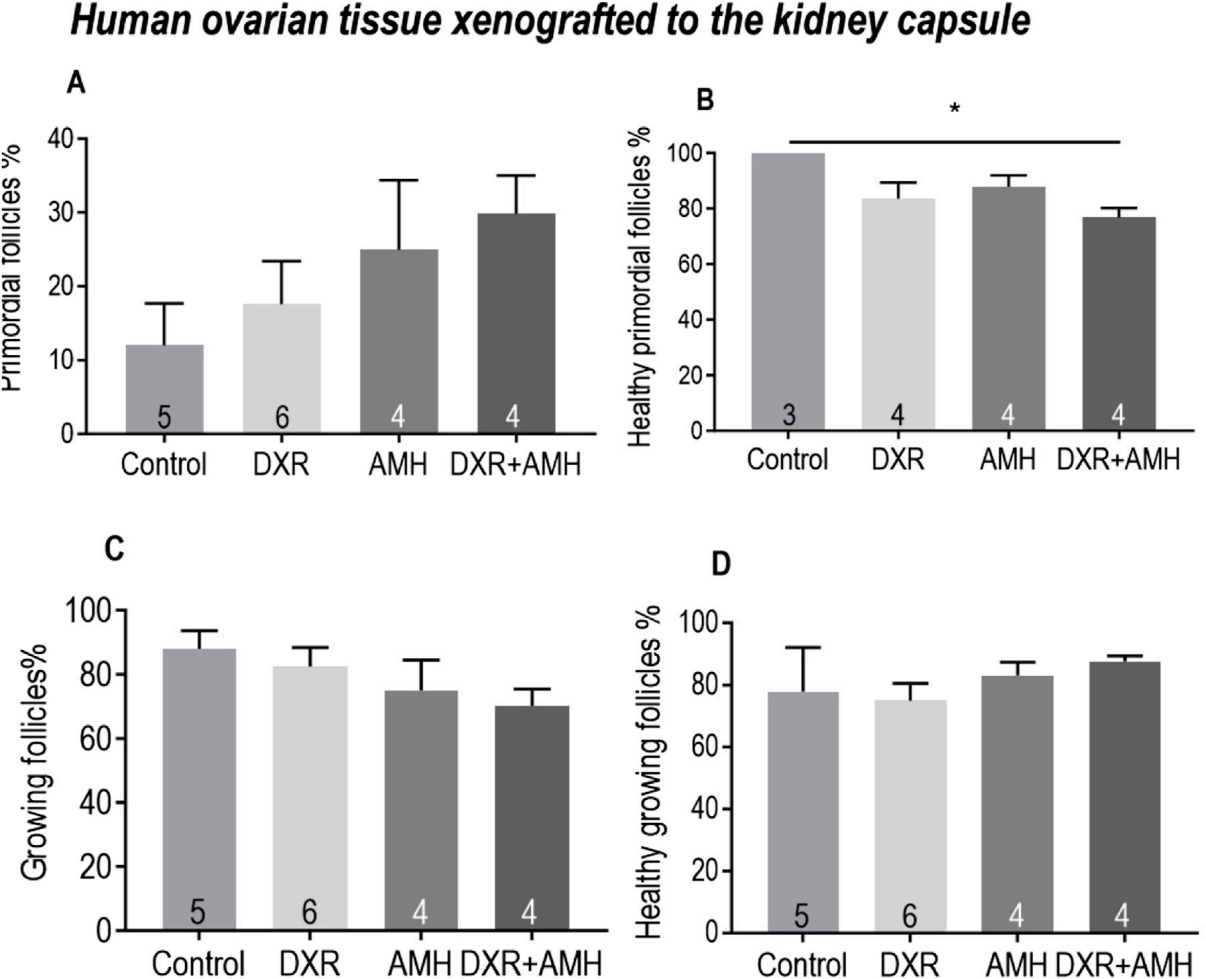
Analysis of follicles in human ovarian tissue xenografts retrieved from beneath the kidney capsule of mice treated with AMH and/or DXR stained with H and E. **(A)** Proportion of follicles that were primordial follicles in each treatment group. **(B)** Proportion of primordial follicles that were morphologically healthy (The sample number is different here. Since two pieces of ovarian tissue in the Control and DXR groups contained only growing follicles without primordial follicles, the ‘n’ in Control and DXR groups in Figure 7B is two fewer than that in Figure 7A. **(C)** Proportion of follicles that were growing. **(D)** Proportion of growing follicles that were morphologically healthy. The data are presented as Mean ± S.E.M. Number in the base of the column is the sample number. *P < 0.05. **P < 0.01. Total follicle numbers (Mean ± S.E.M.): Control 76.7 ± 24.62; AMH 147 ± 48.88; DXR 135.7 ± 42.19; DXR + AMH 131.1 ± 51.75.

For human ovarian tissue xenotransplanted under the kidney capsule, there was no difference in the proportion of primordial follicles between the control, AMH, DXR or co-treatment groups (Figure 7A). There was a significantly lower proportion of healthy primordial follicles between the control and co-treatment groups (Figure 7B). There was no difference in the proportion of total or healthy growing follicles between groups (Figures 7C, D).

**FIGURE 8:**
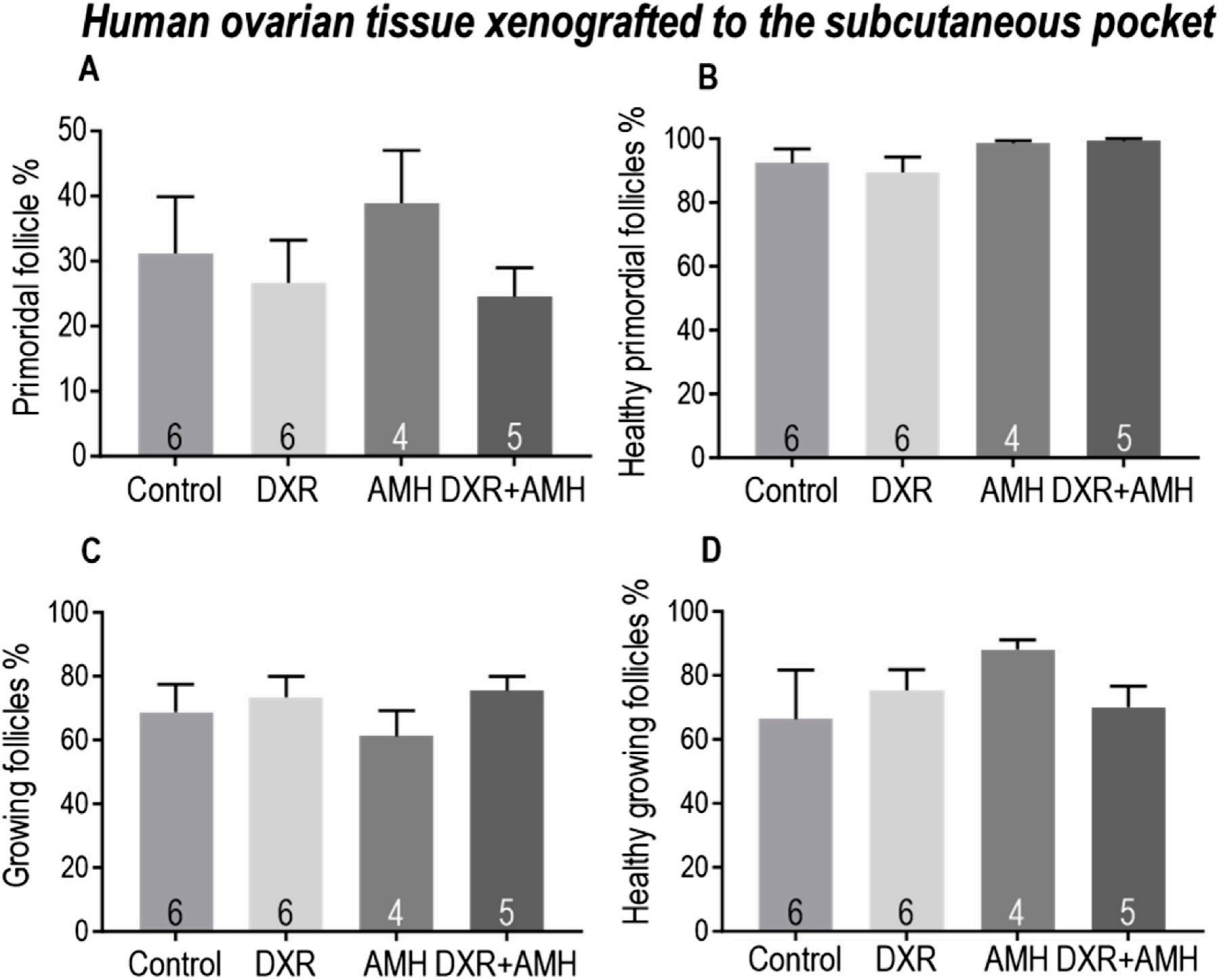
Analysis of follicles in human ovarian tissue xenografts retrieved from a subcutaneous pocket of mice treated with AMH and/or DXR stained with H and E. **(A)** Proportion of follicles that were primordial follicles in each treatment group. **(B)** Proportion of primordial follicles that were morphologically healthy. **(C)** Proportion of follicles that were growing. **(D)** Proportion of growing follicles that were morphologically healthy. The data are presented as Mean ± S.E.M. Number in the base of the column is the sample number. *P < 0.05. Total follicle numbers (Mean ± S.E.M.): Control 278.6 ± 137.6; AMH 511.5 ± 157.5; DXR 330.2 ± 91.73; DXR + AMH 220.4 ± 105.3.

Similar results were observed in human ovarian tissue xenotransplanted subcutaneously, with no significant differences in the proportion of total primordial and growing follicles, nor the proportion of healthy follicles (Figure 8).

There was no difference in the total primordial follicle percentages between the two different xenotransplantation sites in response to different treatments (Supplementary Figure S2). In the co-treatment group, there was a significantly lower percentage of healthy primordial follicles and a higher percentage of unhealthy primordial follicles detected in the xenografts transplanted under the mouse kidney capsule (Supplementary Figure S2B).

**FIGURE 9:**
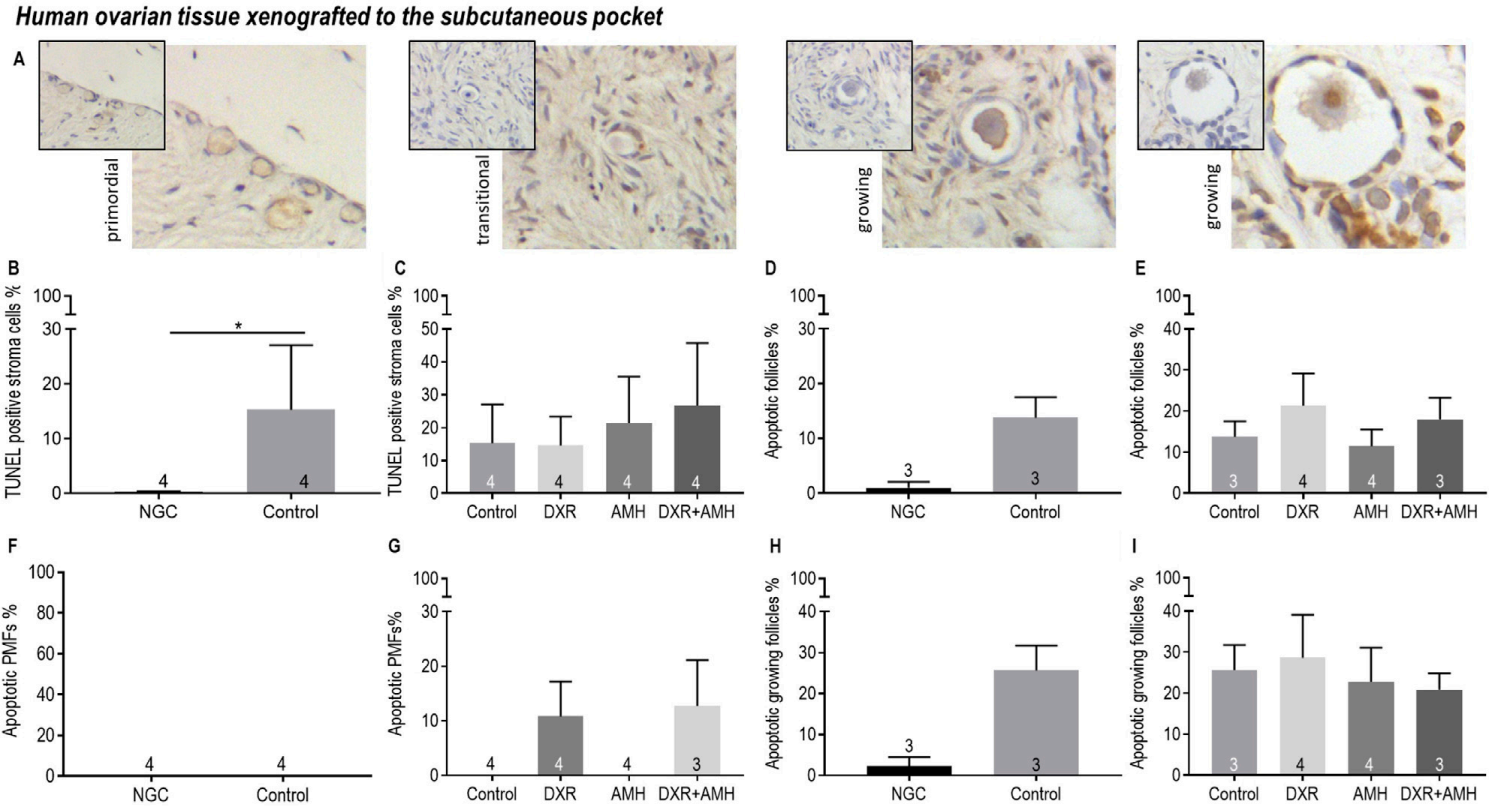
Analysis of apoptotic stroma cells, primordial follicles and growing follicles in non-transplanted human ovarian tissue and xenografts retrieved from a subcutaneous pocket of mice treated with AMH and/or DXR stained with TUNEL. **(A)** Representative images of follicles in human ovarian xenografts stained with TUNEL. **(B)** Proportion of stroma cells that were TUNEL-positive in the non-grafted control ovarian tissue (NGC) and the Control transplanted group. **(C)** Proportion of stroma cells that were TUNEL-positive in tissue retrieved from the control and treated groups. **(D)** Proportion of follicles that were classed as apoptotic in the non-grafted control ovarian tissue (NGC) and the Control transplanted group. **(E)** Proportion of follicles that were classed as apoptotic in tissue retrieved from the control and treated groups. **(F)** Proportion of primordial follicles (PMFs) that were classed as apoptotic in the non-grafted control ovarian tissue (NGC) and the Control transplanted group. **(G)** Proportion of PMF that were classed as apoptotic in tissue retrieved from the control and treated groups. **(H)** Proportion of growing follicles (PMF) that were classed as apoptotic in the non-grafted control ovarian tissue (NGC) and the Control transplanted group. **(I)** Proportion of growing follicles that were classed as apoptotic in tissue retrieved from the control and treated groups. The data are presented as Mean ± S.E.M. Number in the base of the column is the sample number. *P < 0.05.

Apoptosis of stroma cells and follicles in human ovarian xenografts was assessed using the TUNEL assay (Figure 9). There was increased apoptosis in subcutaneously xenotransplanted ovarian stromal cells compared to non-grafted control tissue (Figure 9B). There was no difference in the proportion of apoptotic stromal cells between the different treatment groups (Figure 9C). TUNEL-positive primordial follicles were detected in DXR treated groups, while none were detected in non-DXR-treated groups, however this was not statistically significant (Figure 9G). AMH treatment had no effect on the detection of apoptosis.

## Discussion

In this study we demonstrate that DXR induced primordial follicle loss in pre-pubertal mouse ovarian tissue *in vitro*. When administered as a single IP injection *in vivo*, DXR also induced primordial follicle loss and loss of healthy growing follicles in adult mice, however the proportion of primordial or growing follicles was not affected in xenotransplanted human pre-pubertal ovarian tissue. Human pre-pubertal ovarian tissue was used in this study because DXR is widely used in childhood cancer patients and follicle density is higher in childhood than adulthood. A retrospective study evaluated the follicle density of 830 women aged 4–43 years and found that the follicle density within ovarian cortex was highest at the age of 15 years and then decreased with age (Liebenthron et al., 2019). In human adult ovarian tissue, it might be more difficult to test the effects of DXR due to the lower follicle density. AMH treatment alone did not attenuate primordial follicle loss in mice (*in vitro* and *in vivo*) nor in xenotransplanted human samples. Comparing ovaries treated with 50 ng/mL and 100 ng/mL AMH-treated only, co-treatment with DXR induced significant primordial follicle loss (*p* < 0.05, Figure 3D). However, when exploring a potential protective effect of AMH *in vitro*, lower doses of AMH (50, 100 ng/mL) did not prevent DXR causing a significant drop in follicle numbers, whereas although there is also a decrease in primordial follicle numbers with the higher AMH dose of 200 ng/mL, this decrease was not significant. This suggests that a high dose of AMH may offer a modicum of protection against DXR on primordial follicles. However, more experiments with larger sample sizes and higher doses of AMH will be needed to determine whether AMH has any significant, and more importantly, clinically relevant, ability to rescue DXR-induced primordial follicle loss. In summary, AMH might ameliorate DXR-induced primordial follicle loss in pre-pubertal mouse ovaries but more work is needed to confirm this hypothesis. The effect of AMH against DXR remains unclear in human ovarian tissues. To our knowledge, this is the first study to illustrate there may be an effect of AMH on DXR-induced primordial follicle loss on pre-pubertal mouse ovaries.

DXR has been linked to diminished ovarian reserve, ultimately resulting in POI following treatment. The dosage and duration of chemotherapy treatment is known to influence the risk of developing POI (Chiarelli et al., 1999). We found DXR to cause a reduction in primordial follicle numbers in mice, in line with previous studies (Morgan et al., 2013). In the current study we found that 50 ng/mL DXR was sufficient to cause primordial follicle loss in mouse ovaries *in vitro*, with the concentrations of DXR used (50–200 ng/mL) being within the reported plasma range for cancer patients (20–600 ng/mL; Barpe et al., 2010). A single dose of 5 mg/kg (IP injection) DXR killed mice within 1 week (Denard et al., 2015). There are no deaths reported following a single injection of DXR at a dose of 4 mg/kg so far. Therefore, in consideration of the need for animal survival so the valuable donated human tissue samples were not lost, a dose of DXR at 4 mg/kg was used in our experiment. A single IP injection of 4 mg/kg DXR caused a loss of both primordial and growing follicles in mouse ovaries *in vivo* after 7 days, similar to findings of other studies (Guerreiro et al., 2016; Kano et al., 2017). The functionality of DXR was further confirmed by reduced mouse body and spleen weight, consistent with a previous study (Molyneux et al., 2011). We found decreased proportions of morphologically healthy growing follicles and increased proportions of apoptotic primordial and growing follicles, indicating DXR-induced damage to mouse follicles, consistent with other reports (Roti Roti et al., 2012; Kropp et al., 2015; Herrero et al., 2023). However, we did not find evidence of DXR-induced primordial follicle loss in xenotransplanted pre-pubertal human ovarian tissue. This may be due to the dose used in this study which is the lower end of the human relevant dose of DXR (0–10 mg/kg DXR, equivalent to 8–400 mg/cm^3^ in humans; Scheithauer et al., 1985; Ferguson et al., 1993). Although, more recently, human ovarian tissue cultured for 6 days with 1 μg/mL or 2 μg/mL DXR on day 2 also did not induce significant primordial follicle loss (Lopes et al., 2020).

The mechanism of DXR-induced primordial follicle loss in humans remains poorly understood. Primordial follicle loss after DXR treatment in mice was reported to be due to both direct atresia and accelerated activation (Wang et al., 2019). Direct damage to primordial follicles has also been reported in human ovarian tissue treated with DXR *in vitro* and as xenografts (Soleimani et al., 2011; Lopes et al., 2020). It should be noted that few studies distinguish between primordial and transitional follicles, a distinction that may be critical in evaluating the effects of chemotherapy drugs on the ovarian reserve since transitional follicles may belonging to the growing follicle pool (Braw-Tal, 2002). In our study, TUNEL-positive human primordial follicles were only observed in DXR treated groups (albeit not in significant numbers) indicating possible damage to the health of human primordial follicles. The lack of significance may be due to the lower dose of DXR used or the number of follicles analysed. Previous studies have used various doses with considerable differences between *in vivo* and *in vitro* studies. A single injection of DXR at 10 mg/kg into SCID mice containing xenografted human ovarian cortex led to higher levels of apoptosis in the primordial follicles compared to the control group (Soleimani et al., 2011); far in excess of the single dose of 5 mg/kg of DXR that killed mice within 1 week (Denard et al., 2015). In culture there is clearly more latitude for administering DXR at higher doses. Using a dose of DXR at 1 μg/mL when culturing human adult ovarian cortex did not have a significant effect on follicular health, whereas 2 μg/mL significantly increased the proportion of unhealthy growing follicles compared to controls (Lopes et al., 2020). Thus, in future experiments, although a higher dose of DXR such as 10 mg/kg may be more suitable for assessing the effect of DXR on human follicles in mice hosting human ovarian tissue, there needs to be careful consideration for the lethality of administering DXR at this concentration and study design.

Due to the high proportion of stromal tissue in human ovarian cortex and its important role in supporting follicle growth and development, the effect of DXR on human ovarian stromal cells was also evaluated in this study. There was no detectable effect of DXR at 4 mg/kg on the health of stromal cells within xenografted human ovarian cortex. In an *in vitro* study, DXR did not lead to apoptosis in human ovarian stromal tissue but did reduce stomal cell proliferation (Lopes et al., 2020). In the future, it would be interesting to see whether DXR induce primordial follicle loss through influencing the fate of their surrounding stromal cells in the ovarian cortex.

AMH has been suggested as a potential fertility protective agent, due to its ability to prevent primordial follicle activation and preserve the ovarian reserve (Durlinger et al., 2002; Hayes et al., 2016; Yang et al., 2017). However, studies using human ovarian tissue have been mainly carried out *in vitro*, and results are conflicting (Schmidt et al., 2005; Carlsson et al., 2006; Man et al., 2017). In the current study, although AMH treatment for 6 days of mouse ovary culture resulted in a trend of more primordial follicle numbers than controls, there was no significant effect of AMH on primordial follicle numbers, using concentrations of 50–200 ng/mL; thus, with an increased sample size, this trend may become significant. A recent study showed that 12-day mouse ovaries cultured in 100 ng/mL rhAMH (R&D systems) did not show a different on primordial follicle numbers compared to the control group, but attenuated primordial follicle loss induced by cyclophosphamide (Kashi et al., 2023). Previous studies have reported suppressive effects at higher concentrations of 200–400 ng/mL (recombinant human AMH, 8-day culture; Mark-Kappeler et al., 2010) and 900 ng/mL (recombinant rat AMH, 2-day culture; Durlinger et al., 2002).

Compared to the control group, there was no significant effect of AMH on the number of primordial follicles in mouse ovaries *in vivo*. This is consistent with Kano et al. (2017), who reported that AMH treatment alone for up to 15 days has no effect on the primordial follicle numbers in mouse ovaries *in vivo*. However, in their study, fewer growing follicles were found in the AMH-treated group when AMH administration was extended for 40 days, suggesting a blocking effect of AMH on primordial follicle activation (Kano et al., 2017). However, a decline in growing follicles was not demonstrated in our experiment. This could be attributed to a lower dose used and/or shorter time of AMH administration. Hayes et al. (2016) used the same dose of recombinant mouse AMH as used in the current study (300 ng AMH/day/mouse) for daily IP injections in mice (n = 5) for 28 days, which resulted in a higher percentage of primordial follicles in the AMH group compared to controls. The bioactivity and cross-reactivity of the rhAMH (R&D system) used in the current study had been previously confirmed by another group who tested the phosphorylation of SMAD 1,5,8 which was increased between 3 and 6 h post IP injection of rhAMH (Roness et al., 2019). The bioactivity of rhAMH (R&D system) was also previously confirmed by the regression of mouse Müllerian ducts after exposure to it (Yamamoto et al., 2018). Moreover, the lack of significance in primordial follicle numbers in the co-treatment groups compared to the DXR groups in culture (Figure 4) would indicate the product is bioactive in mouse ovaries.

AMH did not attenuate primordial follicle loss or measurably ameliorate primordial follicle activation (assessed by lack of change in the number of growing follicles) in xenotransplanted human ovarian tissue during this study. This may be due to the significant follicle activation that occurs during xenotransplantation as observed between the non-grafted control and tissue transplanted under the kidney capsule. Detti et al. (2018) found that 7-day pre-treatment of mice with AMH prevented follicle depletion in xenotransplanted pre-pubertal human ovarian tissue. We administered AMH 10 days after xenotransplantation due to enforced limitations on mouse handling post-surgery, which may not be optimal to investigate the effect of AMH since the majority of primordial follicles might have been activated. Therefore, utilising other regimens for AMH administration such as pre-treatment of AMH before xenografting, or utilising osmotic pumps, as well as different doses and durations should be explored in future studies.

We also cannot exclude the possibility that AMH may have a different half-life in different species; i.e., human versus mouse. AMH has been reported possessing an approximately 48-h half-life according to previous studies in human and bovine (Vigier et al., 1983; Griesinger et al., 2012). AMH elimination reaches 95% after 5 days and can be considered complete after 8 days in human (Griesinger et al., 2012). However, Roness et al. (2019) reported that AMH had disappeared from mouse ovaries by 17 h after IP administration *in vivo*. Therefore, for future studies, short interval IP injections of AMH or alternative administration systems such as osmotic pumps might be advantageous since osmotic pumps have resulted in a continuous delivery of AMH in mice (Kano et al., 2017; Detti et al., 2018).

Importantly, when considering administering exogenous AMH as a clinical treatment, in alignment with previous studies (Kong et al., 2016; Detti et al., 2018; Rosario et al., 2024), exogenous AMH did not adversely affect the health of mouse or human ovarian follicles. Moreover, it did not harm stromal cells in human ovarian cortex, and there were no negative effects on mouse body weight or spleen weight.

Interestingly, we find that AMH provided an element of protection against DXR-induced follicle loss in pre-pubertal mouse ovaries *in vitro*, though follicle numbers were still low in DXR-treated ovaries. However, this was not replicated when we administered AMH to adult mice *in vivo* as AMH did not attenuate primordial follicle loss induced by DXR. This is inconsistent with a previous study by Kano et al. (2017), who demonstrated the ovarian protective efficacy of AMH against DXR-induced primordial follicle loss in adult mouse ovaries *in vivo*. The studies differed in both AMH and DXR administration regimen and therefore an effect may not have been observed here due to a smaller doses and shorter administration periods of both AMH and DXR compared to Kano et al. (2017) where adult mice were injected with 14.4 µg/day AMH for 14 days and two injections of DXR at 7.5 mg/kg and 6.0 mg/kg. In the case of another chemotherapy agent, AMH rescued Cyclophosphamide-induced primordial follicle loss in mouse ovarian tissue *in vitro* after cotreatment with Cyclophosphamide metabolite (phosphoramide mustard) and AMH at 100 ng/mL for only 2 h followed by 7 days of culture (Roness et al., 2015), indicating unsurprisingly, that AMH is likely to have different effects with different chemotherapy agents.

Although further insight would be gleaned by knowing more about the regulation of endogenous AMH production and also the pathway for AMH action under the influence of DXR. Previous research found that there was a trend for decreased endogenous AMH 14 days after two weekly DXR treatment (7.5 mg/kg) in mice *in vivo* (Kano et al., 2017) likely due to the loss of growing follicles. They speculated that the decreased endogenous AMH might influence the negative feedback of primordial follicle activation, inducing more primordial follicles to begin to grow, and thus exogenous AMH treatment would preserve the ovarian reserve during chemotherapy. However, if the DXR dose we used (4 mg/kg) did not induce a decrease of endogenous AMH, administration of exogenous AMH might not result in a significant protective effect of AMH against DXR. Further studies exploring how DXR affects endogenous AMH production and expression of AMH receptors would provide insight to this possibility. In addition, DXR has been shown to activate primordial follicles via AKT-FOXO3a pathway (Wang et al., 2019) and thus unravelling the influence of DXR under AMH suppression would be helpful in understanding the role of AMH on primordial follicle activation caused by DXR.

When comparing transplantation sites, the only significant difference we observed was that in the human ovarian tissue xenografted under the mouse kidney capsule rather than the subcutaneous pocket, a lower proportion of healthy primordial follicles was observed in the co-treatment group. This might be due to the fact that the kidney has better vascularization so relatively more DXR is delivered there acting negatively to the tissue compared to the subcutaneous pocket where an extensive vascular supply needs to be established.

## Conclusion

AMH has been proposed as a candidate fertility protective agent for preventing chemotherapy-induced POI. According to our knowledge, this is the first study to investigate the effectiveness of recombinant human AMH against DXR on xenografted pre-pubertal human ovarian tissue and prepubertal mouse ovarian tissue. Administration of rhAMH to mice appears safe with no detectable negative effects on follicle health in either mouse or human ovaries, or stromal cells in human ovarian cortex. With the dose and administration period of AMH and DXR we used, we found no evidence that AMH could attenuate primordial follicle loss in mouse ovaries *in vivo* or in human ovarian xenografts during DXR treatment. Previously there was only one study (Kano et al., 2017) that has demonstrated that AMH prevented DXR-induced primordial follicle loss and this was in adult mice *in vivo* using a different administration regimen. Therefore, future experiments in mice hosting human ovarian tissue should explore different dose of both DXR and AMH to conclusively ascertain the effectiveness of AMH as a protective agent for human follicles during DXR administration.

This is the first study to demonstrate that in prepubertal mouse ovaries co-cultured with a higher dose of AMH, DXR did not cause a significant loss in primordial follicles. However, the small sample size accompanied by considerable variation means it would be naïve to rely on these data without interrogation into the validity of this effect. Moreover, this effect, if substantiated, is moderate and thus may not be clinically relevant. Therefore, more experiments with higher/different doses of AMH and large sample size are urgently needed.

Regarding the human ovarian xenografts, because there was no effect of DXR on primordial follicle numbers, the effect of AMH on the prevention of DXR-induced primordial follicle loss was not able to be elucidated in this study. Again, this could be due to the limitations regarding AMH administration and also the half-life of AMH as discussed. Finally, AMH has recently been shown to attenuate Cyclophosphamide-induced phosphorylated FOXO3A expression (Rosario et al., 2024) and we should determine whether AMH has a similar effect with DXR.

Thus, to obtain robust evidence about the potential of AMH in fertility preservation during chemotherapy treatment, alternative strategies and regimens for AMH administration need to be explored in conjunction with DXR administration to fully interrogate the effect of DXR and AMH on human xenografted tissues.

## Data Availability

The raw data supporting the conclusions of this article will be made available by the authors, without undue reservation.

## Supplementary material

SUPPLEMENTARY TABLE S1 Number of retrieved pre-pubertal human ovarian xenografts used for histological analyses (follicle percentages) in this study.

SUPPLEMENTARY FIGURE S1 Effects of DXR on mouse body weight, spleen weight and ovarian volume. **(A)** Mouse weight at the time of the surgery and during 8 days of treatment with PBS, AMH, DXR or co-treatment (DXR + AMH) starting 10 days after surgery. **(B)** The spleen weight of the mice after 8 days of treatment with PBS, AMH, DXR or co-treatment (DXR + AMH). **(C)** The ovarian volume of the mice after 8 days of treatment with PBS, AMH, DXR or co-treatment (DXR + AMH). Data are presented as Mean ± S.E.M. Number in the base of the column is the sample number. *P < 0.05. **P < 0.01. ***P < 0.001. ****P < 0.001.

SUPPLEMENTARY FIGURE S2 Comparison of primordial and growing follicle percentages in the human ovarian xenografts retrieved from the kidney capsule and subcutaneous pocket stained with H and E. **(A)** Total primordial follicle percentages in the human ovarian xenografts retrieved from different groups. **(B)** Healthy primordial follicle percentages in the human ovarian xenografts retrieved from different groups. **(C)** Total primordial follicle percentages in the human ovarian xenografts retrieved from different groups. **(D)** Healthy growing follicle percentages in the human ovarian xenografts retrieved from different groups. The data are presented as Mean ± S.E.M. Number in the base of the column is the sample number. **P < 0.01. Total follicle numbers in grafts under mouse kidney capsule (Mean ± S.E.M.): Control 76.7 ± 24.62; AMH 147 ± 48.88; DXR 135.7 ± 42.19; DXR + AMH 131.1 ± 51.75. Total follicle numbers in subcutaneous grafts (Mean ± S.E.M.): Control 278.6 ± 137.6; AMH 511.5 ± 157.5; DXR 330.2 ± 91.73; DXR + AMH 220.4 ± 105.3.
