# Supplementary material for: Effect of AMH on primordial follicle populations in mouse ovaries and human pre-pubertal ovarian xenografts during doxorubicin treatment
Source: Front Cell Dev Biol. 2024 Aug 27;12:1449156. doi: 10.3389/fcell.2024.1449156 (PMC11383774; doi:10.3389/fcell.2024.1449156)
Supplement: Supplementary file 1 [file Table1.DOCX]

**Table S1.**

| **Sites** | **Control** | **AMH*** | **DXR** | **DXR+AMH** |
| --- | --- | --- | --- | --- |
| Kidney capsule | 5**/6 | 4/6 | 6/6 | 4/4 |
| Subcutaneous | 6/6 | 4/6 | 6/6 | 5/5 |

**Two pieces of tissue from two patients in this group together with the non-grafted ovarian tissue looked abnormal (no nuclear was stained after H & E staining) and were therefore excluded.*

***One piece contained zero primordial and growing follicles, the percentage analyses were therefore not able to be performed.*
