# Supplementary figures and images for: Effect of AMH on primordial follicle populations in mouse ovaries and human pre-pubertal ovarian xenografts during doxorubicin treatment
Source: Front Cell Dev Biol. 2024 Aug 27;12:1449156. doi: 10.3389/fcell.2024.1449156 (PMC11383774; doi:10.3389/fcell.2024.1449156)

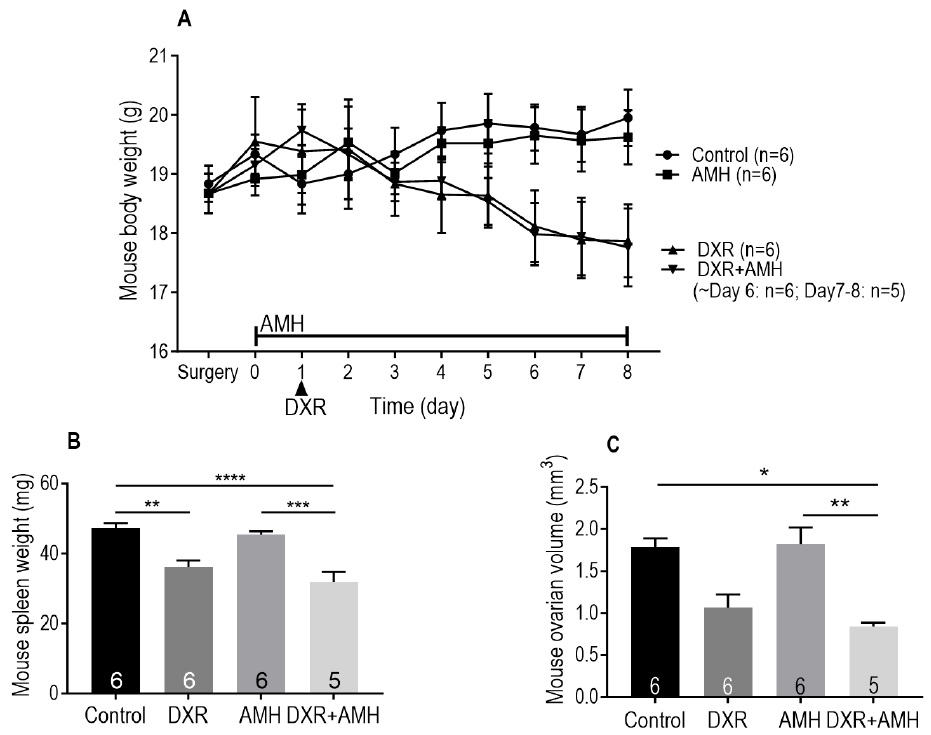

Supplement: Supplementary file 2 [file Image1.JPEG]

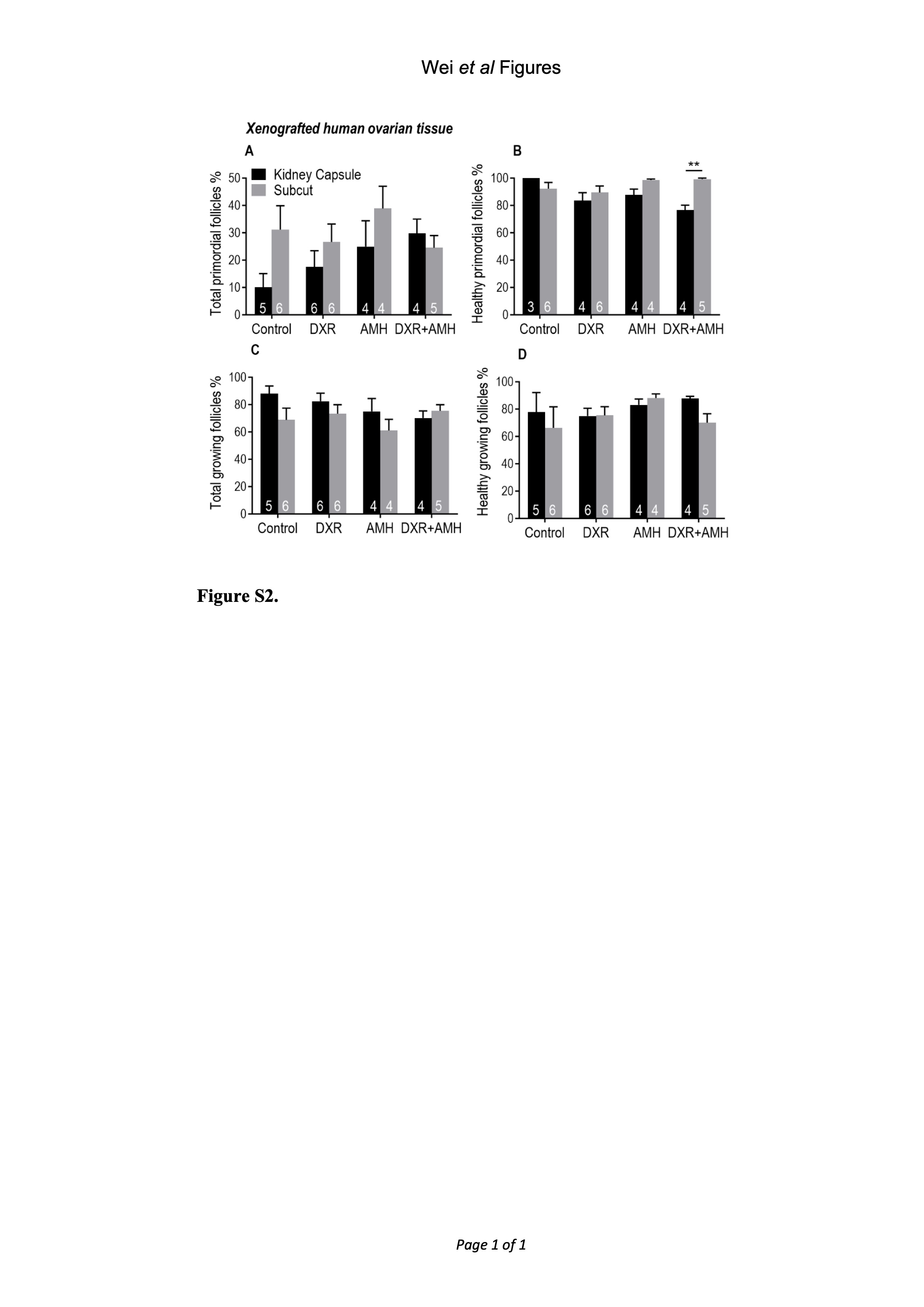

Supplement: Supplementary file 3 [file Image2.JPEG]
